# *Plasmodium falciparum* genetic diversity and multiplicity of infection among asymptomatic and symptomatic malaria-infected individuals in Uganda

**DOI:** 10.1186/s41182-024-00656-7

**Published:** 2024-11-14

**Authors:** Alex Mwesigwa, Moses Ocan, Bryan Cummings, Benson Musinguzi, Shahid Kiyaga, Steven M. Kiwuwa, Stephen Okoboi, Barbara Castelnuovo, Everd Maniple Bikaitwoha, Joan N. Kalyango, Charles Karamagi, Joaniter I. Nankabirwa, Samuel L. Nsobya, Pauline Byakika-Kibwika

**Affiliations:** 1 Clinical Epidemiology Unit, School of Medicine, College of Health Sciences, Makerere University, P. O. Box 7072, Kampala, Uganda; 2 Department of Microbiology and Immunology, School of Medicine, Kabale University, P. O Box 314, Kabale, Uganda; 3 Department of Pharmacology & Therapeutics, School of Biomedical Sciences, College of Health Sciences, Makerere University, P.O. Box 7072, Kampala, Uganda; 4 Malaria Research Program, Center for Vaccine Development and Global Health, University of Maryland School of Medicine, 655 W. Baltimore St, Baltimore, MD 21201 USA; 5 Departent of Medical Laboratory Science, Faculty of Health Sciences, Muni University, P.O Box 725, Arua, Uganda; 6 Department of Immunology and Molecular Biology, School of Biomedical Sciences, College of Health Sciences, Makerere University, P.O. Box 7072, Kampala, Uganda; 7 Department of Biochemistry, School of Biomedical Sciences, College of Health Sciences, Makerere, University, P.O. Box 7072, Kampala, Uganda; 8 Infectious Diseases Institute, College of Health Sciences, Makerere University, P. O. Box 7072, Kampala, Uganda; 9 Department of Community Health, School of Medicine, Kabale University, P. O Box 314, Kabale, Uganda; 10 Infectious Diseases Research Collaboration, College of Health Sciences, Makerere University, P.O. Box 7072, Kampala, Uganda; 11 Department of Medicine, School of Medicine, College of Health Sciences, Makerere University, P. O. Box 7072, Kampala, Uganda; 12 Mbarara University of Science and Technology, Mbarara, Uganda

**Keywords:** *P. falciparum*, Genetic diversity, Multiplicity of infection, Malaria, Uganda

## Abstract

**Background:**

*Plasmodium falciparum* (*P. falciparum*) remains a significant public health challenge globally, especially in sub-Saharan Africa (SSA), where it accounts for 99% of all malaria infections. The outcomes of *P. falciparum* infection vary, ranging from asymptomatic to severe, and are associated with factors such as host immunity, parasite genetic diversity, and multiplicity of infection (MOI). Using seven neutral microsatellite markers, the current study investigated *P. falciparum* genetic diversity and MOI in both asymptomatic and symptomatic malaria individuals in Uganda.

**Methods:**

This cross-sectional study analyzed 225 *P. falciparum* isolates from both asymptomatic and symptomatic malaria patients, ranging in age from 6 months to ≥ 18 years. *P. falciparum* genetic diversity, MOI, and multi-locus linkage disequilibrium (LD) were assessed through genotyping of seven neutral microsatellite markers: Poly-α, TA1, TA109, PfPK2, 2490, C2M34–313, and C3M69–383. Genetic data analysis was performed using appropriate genetic analysis software.

**Results:**

*P. falciparum* infections exhibited high genetic diversity in both asymptomatic and symptomatic individuals. The mean expected heterozygosity (He) ranged from 0.79 in symptomatic uncomplicated malaria cases to 0.81 in asymptomatic individuals. There was no significant difference (*p* = 0.33) in MOI between individuals with asymptomatic and symptomatic infections, with the mean MOI ranging from 1.92 in symptomatic complicated cases to 2.10 in asymptomatic individuals. Polyclonal infections were prevalent, varying from 58.5% in symptomatic complicated malaria to 63% in symptomatic uncomplicated malaria cases. A significant linkage disequilibrium (LD) was observed between asymptomatic and symptomatic uncomplicated/complicated infections (*p* < 0.01). Genetic differentiation was low, with F_ST_ values ranging from 0.0034 to 0.0105 among *P. falciparum* parasite populations in asymptomatic and symptomatic uncomplicated/complicated infections.

**Conclusion:**

There is a high level of *P. falciparum* genetic diversity and MOI among both symptomatic and asymptomatic individuals in Uganda. Asymptomatic carriers harbor a diverse range of parasites, which poses challenges for malaria control and necessitates targeted interventions to develop effective strategies.

**Supplementary Information:**

The online version contains supplementary material available at 10.1186/s41182-024-00656-7.

## Background

*Plasmodium falciparum* (*P. falciparum*) malaria poses a significant global health challenge. Among the five parasite species causing human malaria, *P. falciparum* is responsible for the greatest burden of morbidity and mortality, particularly in sub-Saharan Africa (SSA), where it accounts for 99% of the cases [1]. *P. falciparum* infection manifests in a range of clinical outcomes, from asymptomatic parasitemia to symptomatic uncomplicated (mild) and complicated (severe) disease [2, 3]. These manifestations are influenced by various factors, including host factors such as age and immunity, transmission intensity, and parasite-related factors such as genetic diversity, multiplicity of infection (MOI), and parasite density [4–6].

*P. falciparum* possesses a haploid genome spread across 14 chromosomes, totaling 23 megabases (Mb). The genome encodes multigene families, such as var, rif, and stevor genes, which are crucial for invading host red blood cells [7, 8]. These genes encode hypervariable antigens and virulence factors that are exported to the surface of the infected erythrocyte and are targeted by naturally acquired immunity [8, 9]. They are expressed haplotypically, with only one copy per infection, allowing parasites to maintain fitness through gene switching.

Novel *P. falciparum* genotypes arise from genetic recombination or haplotype switching during the parasite lifecycle in the female Anopheles mosquito vector [10, 11]. In humans, genetically complex infections may occur due to exposure to multiple parasite strains from different mosquito bites (superinfection) or from a single mosquito bite carrying multiple strains (co-transmission) [12, 13]. Multiple *P. falciparum* genotypes result in a higher MOI [14] and may lead to an increased release of proinflammatory cytokines such as IL-6 and IL-8, contributing to the development of severe malaria [15, 16]. Conversely, immunity to a specific parasite clone can protect against clinical malaria caused by that clone [17]. For example, immunity to *P. falciparum* var gene-encoded *P. falciparum* erythrocyte membrane protein 1 (PfEMP1) is thought to protect individuals against severe malaria infections [18, 19]. Increased host immunity to infections genetically similar to previous infections may protect against symptomatic malaria [20]. Conversely, *P. falciparum* infection following recovery from previous polygenic infections is less likely to progress to clinical disease [21, 22], whereas infection with new parasite strains increases the risk of symptomatic infections [23] and may favor gametocyte development [24, 25], enhancing parasite transmissibility [26].

The evaluation of *P. falciparum* genetic diversity and MOI in resource-limited settings typically relies on targeted genotyping of antigen-coding loci such as *msp-1*, *msp-2*, and *glurp* [27, 28]. Although genotyping these antigen-coding loci is readily available, these markers are subject to immune selection [29]. Advanced approaches, such as targeted deep sequencing, are sensitive methods for assessing parasite diversity but remain inaccessible for most laboratories in SSA due to their high development and maintenance costs [30]. Microsatellite markers, considered selection-neutral and not targets for immune evasion, provide an unbiased view of parasite genetic diversity [31]. Neutral polymorphic microsatellites, including Poly-α, TA1, TA109, and PfPK2, offer valuable and cost-effective tools for evaluating *P. falciparum* genetic diversity [32–34]. In the *P. falciparum* genome, these microsatellite loci are abundant, existing as [TA]n, [T]n, and [TAA]n repeats [35]. Their high variability allows them to distinguish between different parasite strains, which is crucial for assessing MOI, an indicator of the number of distinct strains infecting a host. Additionally, their broad genomic distribution provides a comprehensive understanding of genetic diversity across different regions of the genome.

In areas with low malaria transmission, parasite strains exhibit strong linkage disequilibrium (LD), low genetic diversity, and significant population differentiation. Conversely, in high transmission areas, parasite strains show weak LD, high genetic diversity, and minimal population differentiation [33]. Analyzing the *P. falciparum* genetic profile associated with malaria infection enhances understanding of infection severity, risk for antimalarial resistance, and potential effects on treatment outcomes [36, 37]. The genetic diversity and MOI of *P. falciparum* are correlated with malaria severity and are greater in symptomatic infections [38–40] than in asymptomatic infections [41]. However, other studies have reported high genetic diversity and MOI in both asymptomatic and symptomatic malaria infections [23, 42–44], although some have found no significant differences [45]. The diversity of *P. falciparum* in asymptomatic malaria poses challenges for parasite elimination, as asymptomatic infections are often untreated and serve as reservoirs of transmission [46, 47].

In Uganda, studies on *P. falciparum* genetic diversity and MOI have primarily focused on symptomatic children, who often experience high clinical disease rates [34, 48, 49], with little attention given to asymptomatic and adults individuals. This study aimed to evaluate the genetic diversity and MOI of *P. falciparum* infections among both asymptomatic and symptomatic individuals by genotyping seven neutral microsatellite markers.

## Methods

### Study design and population

This was a cross-sectional study. *P. falciparum* genotyping was conducted using dried blood spot (DBS) filter paper samples collected from participants enrolled in cohort studies under the Program for Resistance, Immunology, and Modeling of Malaria (PRISM) project. Details of these cohort studies are described elsewhere [50]. Briefly, each cohort included participants aged 6 months to ≥ 18 years from 100 randomly selected households in three sub-counties: Walukuba (Jinja district), Kihihi (Kanungu district), and Nagongera (Tororo district) from 2011 to 2016. Although malaria transmission may have changed recently, at the time of the study, Walukuba was a relatively low-transmission peri-urban area near Lake Victoria in the south-central part of the country. Kihihi, on the other hand, is a rural area with moderate transmission intensity, bordering Bwindi Impenetrable National Park in the southwestern part of the country. Nagongera is a rural area with high transmission intensity in the southeastern part of the country, near the border with Kenya (Additional file 1, Figure S1). The DBS samples analyzed in the current study were collected from participants on the day of enrollment for parasite detection.

### Participants’ demographics and clinical data

Demographic and clinical data, including age, sex, axillary temperature, hemoglobin levels, and parasite density, were extracted from the PRISM cohort database and managed in an Excel spreadsheet. Participants’ hemoglobin levels were systematically measured using a portable spectrophotometer (Hemocue).

### Laboratory methods

Laboratory assays, including malaria microscopy and parasite genotyping, were conducted at the Molecular Research Laboratory at the Infectious Disease Research Collaboration in Kampala-Uganda.

### *P. falciparum* parasite density determination

Microscopy slides for parasite density and species detection were prepared using a 10% Giemsa solution and stained for 30 min, with thick and thin blood films used, respectively. Experienced microscopists examined the stained slides under a light microscope at 100 × oil immersion. Parasite density of *P. falciparum* was assessed by counting asexual parasites against 200 leukocytes. The parasite density per µL of blood was calculated by multiplying the total parasite count by 40, assuming an average of 8000 leukocytes per µL of blood [51]. For quality control, each smear was independently read by two microscopists. Discrepancies, defined as differences in species diagnosis, parasite density > 50%, or presence of parasites, prompted a review by a third microscopist. Final parasitemia was determined by averaging the readings of the two microscopists or, in cases of disagreement, by averaging the third microscopist’s reading with the closest of the initial two. The third microscopist’s reading was used as the final determination for parasite species.

### P. falciparum DNA extraction and detection

The genomic DNA of *P. falciparum* was extracted from DBS filter paper samples using Chelex 100 Resin (Sigma‒Aldrich, USA) following the method described by Bereczky et al. [52]. Briefly, each filter paper punch (6 mm disc) was incubated overnight at 4 °C in 1 mL of 0.5% saponin in phosphate-buffered saline (PBS). The discs were washed for 30 min in PBS at 4 °C, transferred to new tubes containing 25 μL of stock solution (20% Chelex-100 and 75 μL of distilled water), and vortexed for 30 s. The tubes were then heated at 99 °C for 15 min to elute the DNA, vortexed again, and centrifuged at 10,000 × *g* for 2 min. The extracted DNA was stored at − 20 °C until further use. Detection and confirmation of *P. falciparum* were performed through genotyping of *P. falciparum* 18S rRNA using nested PCR [53].

### Microsatellite genotyping

Parasite genotyping utilized seven neutral polymorphic microsatellites distributed across six chromosomes of *P. falciparum*: Poly-α on chromosome 4, TA1 and TA109 on chromosome 6, PfPK2 on chromosome 12, 2490 on chromosome 10, C2M34–313 on chromosome 2, and C3M69–383 on chromosome 3, following established methods. The PCR reactions for each marker were conducted in a total volume of 15 µL. Poly-α, TA1, TA109, PfPK2, and 2490 microsatellites were nested, whereas C2M34–313 and C3M69–383 microsatellites were unnested [54, 55]. For the nested PCR reactions, the primary reaction for each marker was carried out in a 15 µL solution containing 10.5 µL of molecular-grade PCR water, 1.5 µL of 10 × reaction buffer, 0.3 µL of dNTPs (1.25 mM), 0.3 µL of Forward Primer (10 μM), 0.3 µL of Reverse Primer (10 μM), 0.25 µL of AmpliTaq Gold (5 U/μL), and 2 µL of DNA template. The Round 1 PCR conditions were: 94 °C for 2 min, followed by 25 cycles of (94 °C for 30 s, 42 °C for 30 s, 40 °C for 30 s, 65 °C for 40 s), and ending with 65 °C for 2 min. The secondary reaction contained the same reagents as the primary reaction, with the addition of 0.3 µL of the labeled primer for each marker. A 2 µL sample of the primary reaction product was used in a final volume of 15 µL for the nested PCR reactions. The Round 2 PCR conditions were: 94 °C for 2 min, followed by 25 cycles of (94 °C for 20 s, 45 °C for 20 s, 65 °C for 30 s), and ending with 65 °C for 2 min. PCR conditions for the C2M34–313 and C3M69–383 microsatellites were as follows: 94 °C for 2 min, followed by 5 cycles of (94 °C for 30 s, 50 °C for 30 s, 60 °C for 30 s), 40 cycles of (94 °C for 30 s, 45 °C for 30 s, 60 °C for 30 s), and ending with 60 °C for 2 min (Additional file 2, Table S2). A 2 µL sample of the PCR product was then electrophoresed on a 2% agarose gel to confirm amplification. The amplified PCR products were transferred to safe-lock DNA amplicon storage tubes, securely wrapped in aluminum foil, and sent to Inqaba Biotec in South Africa for microsatellite fragment analysis using an ABI capillary electrophoresis platform.

### Microsatellite analysis

An ABI 3730xl (Thermo Fisher/Hitachi) genetic analyzer was used to determine the lengths of the microsatellite fluorescence-labeled PCR products. GeneMarker HID V2.9.5 software was used to score the peaks. For samples producing more than one peak, the highest peak was defined as the dominant allele, while other peaks were defined as minor alleles if their peak heights were > 200 relative fluorescence units (RFU) and > 20% of the highest peak.

### Data analysis

The participants’ demographic and clinical data, including age, gender, parasite density, and hemoglobin levels, were managed in Excel and exported to STATA version 17.0 (Stata Corp., College Station, TX, USA) for analysis. Statistical comparisons of axillary temperature, hemoglobin levels, and parasite density among asymptomatic, symptomatic uncomplicated, and symptomatic complicated cases were performed using the Kruskal–Wallis test. The statistical significance threshold was set at *p* < 0.05. Microsatellite data were retrieved from the ABI 3730xl genetic analyzer (Thermo Fisher/Hitachi). Subsequent genetic analysis was conducted on a total of 211 samples, where at least five microsatellite markers were successfully amplified. Among these 211 successfully amplified samples, the majority (129, 61%) were polyclonal infections. Therefore, all genetic analyses were conducted using only the predominant alleles to minimize bias associated with analyzing samples with multiple infections.

### *P. falciparum* genetic diversity

Genetic diversity of *P. falciparum*, reflecting variation due to recombination or mutation [56], was assessed by calculating the mean number of alleles (A), the number of effective alleles (Ne), and the mean expected heterozygosity across each locus using GENALEX 6.5 software [57]. Expected heterozygosity (He), defined as the probability that two randomly selected individuals carry distinct alleles at a marker locus, was calculated using the following formula:

$${\text{He }} = \left[ {{\text{n}}/\left( {{\text{n}} - 1} \right)} \right]\left[ {1 - \sum {{\text{pi}}^\wedge2} } \right], $$

where ‘n’ is the number of isolates analyzed and ‘pi’ is the frequency of the ith allele in the population [33].

### *P. falciparum* multiplicity of infection

*P. falciparum* MOI was defined as the number of distinct parasite genotypes coexisting within a given infection [58]. Isolates with one allele were considered monoclonal infections, whereas those with more than one allele were considered polyclonal [59].

### Analysis of multi-locus linkage disequilibrium and genetic differentiation

Multi-locus linkage disequilibrium (LD) measured as the standardized index of association (I_A_^S^) was calculated using the program LIAN version 3.5 [60] for the whole dataset. This index was calculated using the formula:

$$ {\text{I}}_{{\text{A}}}^{{\text{S}}} { } - { } = { }(1/{\text{n}} - 1(\left( {{\text{VD}}/\left( {{\text{VE}}} \right) - 1} \right), $$

where VE is the expected variance of the nth number of loci for which two individuals differ. VD is the observed variance. The significance of the I_A_^S^ values was tested using the Monte Carlo method. Genetic differentiation was assessed using Wrights fixation index (F_ST_) calculated using Arlequin 3.11 [61]. The F_ST_ values ranging from 0 to 0.05 indicates low genetic variability, 0.05–0.15 indicates moderate genetic variability, 0.15–0.25 indicated high great genetic differentiation and > 0.25 indicates substantial genetic differentiation [62].

## Results

### Study population

A total of 225 isolates were genotyped, of which 211 (93.8%) were successfully genotyped on at least five neutral microsatellites and included in the final analysis. Among the successfully genotyped samples, 109 were from males. The age of the participants ranged from 6 months to 53 years, with the majority being less than 11 years old. Parasite density ranged from 16 to 1,600,000 parasites/µL. Significant differences in axillary temperature, parasite density, and hemoglobin levels were detected among asymptomatic, symptomatic uncomplicated, and symptomatic complicated malaria individuals (*p* < 0.05, Kruskal–Wallis test). Symptomatic complicated individuals had the highest parasite density and the lowest hemoglobin levels (Table 1).

**Table 1:** Demographic and clinical characteristics of the study participants

Characteristic	Asymptomatic malaria infection (*n* = 70)	Uncomplicated malaria infection (*n* = 86)	Complicated malaria infection (*n* = 55)	*P*. value
Age (years): < 5	19	39	36	
5–11	42	40	18	
> 18	9	7	1	
Gender ratio (male/female)	34/36	48/38	27/28	
Axillary temperature, ^0^c (mean ± SD)	36.8 ± 0.3	38.6 ± 0.9	38.3 ± 1.1	< 0.001
Mean parasite density/µL (mean ± SD)	5862.5 ± 508.4	61,036.4 ± 671.9	446,303.6 ± 9136.5	< 0.001
Mean Hb g/dL (mean ± SD)	11.5 ± 1.6	11.4 ± 1.9	9.8 ± 2.4	< 0.001

### *P. falciparum* genetic diversity among asymptomatic and symptomatic uncomplicated and complicated study participants

**Fig. 1:**
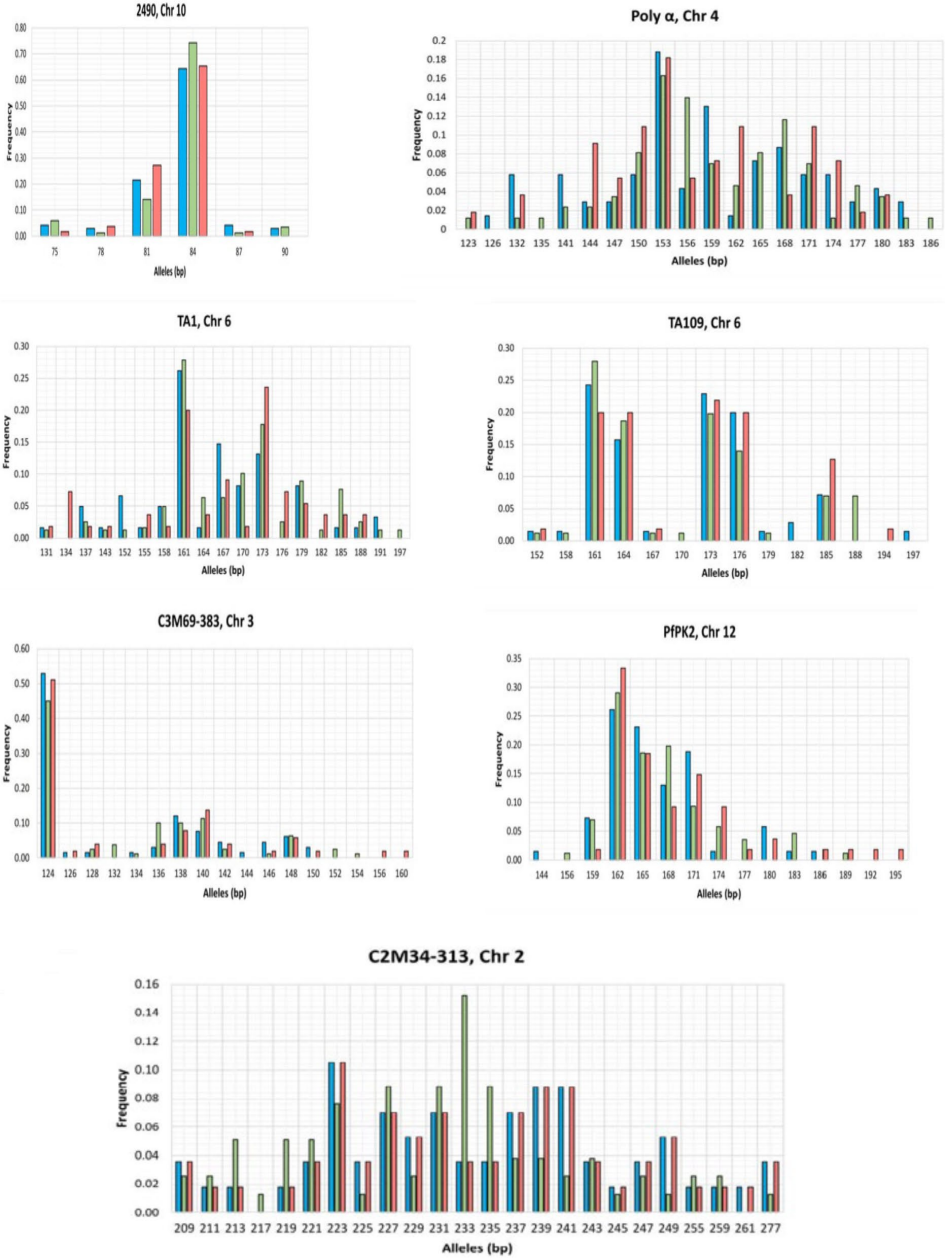
The allele frequencies of the genotyped markers assessed among individuals with asymptomatic (blue), symptomatic uncomplicated (green) and complicated (red) malaria infections. The vertical axis represents the frequencies of individual alleles, whereas the horizontal axis represents the base pairs of each allele. *Chr* chromosome, *bp* base pair

The genetic diversity of *P. falciparum*, as indicated by the number of alleles, allele frequencies, number of effective alleles, and expected heterozygosity, was high across both symptomatic and asymptomatic malaria infections. The Kruskal–Wallis test showed no significant differences in the observed number of alleles, allele frequencies, number of effective alleles, and expected heterozygosity between symptomatic and asymptomatic malaria infections (*p* > 0.05). The most polymorphic microsatellite loci were C2M34–313, TA1, and Poly-α, with 17–23, 15–16, and 14–19 distinct alleles, respectively. The least polymorphic microsatellite locus was 2490, with 5 to 6 distinct alleles (Fig. 1 and Table 2).

**Table 2:** The genetic diversity of *P. falciparum* isolates based on seven neutral microsatellites markers

Locus	Asymptomatic malaria (*n* = 70)			Symptomatic uncomplicated malaria (*n* = 86)			Symptomatic complicated malaria (*n* = 55)		
	Na	Ne	He	Na	Ne	He	Na	Ne	He
2490	6	2.15	0.54	6	1.74	0.43	5	0.14	0.50
Poly α	17	11.15	0.92	19	10.97	0.92	14	10.19	0.92
C2M34–313	23	17.01	0.96	23	14.35	0.94	17	10.09	0.92
TA1	15	7.52	0.88	16	6.96	0.87	16	7.94	0.89
TA109	11	5.47	0.83	11	5.51	0.83	8	5.41	0.83
C3M69–383	12	3.20	0.69	14	4.11	0.77	12	3.39	0.72
PfPK2	10	5.44	0.83	10	5.59	0.83	12	5.32	0.83
Mean	13.43	7.42	0.81	14.14	7.03	0.79	12.29	6.07	0.80
SD	5.50	5.15	0.15	5.76	4.28	0.17	4.28	3.65	0.15

Na = number of different alleles; Ne = number of effective alleles = 1/(∑pi^2); He = unbiased diversity/expected heterozygosity = [n/(n − 1)] [1 − ∑pi^2], and SD = standard deviation. Na, Ne, and He were calculated from the predominant allele dataset. The 95% confidence interval (CI) was calculated as CI = x̄ ± 1.96(SD/√n), where x̄ is the sample mean, SD is the standard deviation of the sample, and n is the sample size

### Expected heterozygosity and number of effective alleles

The mean expected heterozygosity (He) ranged from 0.79 (± 0.17) in individuals with symptomatic uncomplicated malaria to 0.81 (± 0.15) in those with asymptomatic malaria. The number of effective alleles (Ne) ranged from 6.07 (± 3.65) among individuals with complicated malaria to 7.42 (± 5.15) in asymptomatic individuals (Table 2).

### *P. falciparum* MOI among asymptomatic, symptomatic uncomplicated and complicated malaria-infected individuals

The mean MOI was not significantly different (*p* = 0.33) across the various *P. falciparum* infection categories. The mean MOI ranged from 1.92 (± 0.42) in individuals with symptomatic complicated malaria to 2.10 (± 0.36) among asymptomatic malaria individuals (Table 3). The percentages of polyclonal infections were generally consistent across the different *P. falciparum* infection types, ranging from 58.5% (95% CI 44.60–72.39) among individuals with symptomatic uncomplicated malaria to 63% (95% CI 51.22–74.78) among those with complicated malaria.

**Table 3:** *P. falciparum* MOI in asymptomatic, symptomatic uncomplicated, and symptomatic complicated individuals

Locus	Asymptomatic malaria (*n* = 70)				Uncomplicated malaria (*n* = 86)				Complicated malaria (*n* = 55)			
	Number positive	Percentage monoclonal	Percentage polyclonal	Mean MOI	Number positive	Percentage monoclonal	Percentage polyclonal	Mean MOI	Number positive	Percentage monoclonal	Percentage polyclonal	Mean MOI
2490	70	64.3	35.7	1.5	85	64.7	35.3	1.4	55	63.6	36.4	1.4
Poly a	69	42.0	58.0	2.4	86	43.0	57.0	2.2	55	61.8	38.2	1.6
C2M34–313	57	43.9	56.1	1.7	79	48.1	51.9	1.8	52	50.0	50.0	1.7
TA1	62	29.0	71.0	2.1	79	25.3	74.7	2.3	55	10.9	89.1	2.6
TA109	70	47.1	52.9	2.2	85	38.8	61.2	2.2	55	50.9	49.1	2.0
C3M69–383	66	13.6	86.4	2.5	80	12.5	87.5	2.5	51	23.5	76.5	2.3
PfPK2	69	29.0	71.0	2.2	86	26.7	73.3	2.3	54	29.6	70.4	2.1
Mean		38.4	61.6	2.10		37.0	63.0	2.08		41.5	58.5	1.92
SD		16.24	16.24	0.36		17.19	17.19	0.37		20.26	20.26	0.42

The number positive represents the number of samples successfully amplified by each locus. The percentage of monoclonal infections is the proportion of isolates with only one allele score per locus. The percentage of polyclonal infections is the proportion of isolates with more than one allele score per locus

### Linkage disequilibrium (LD) and genetic differentiation

A multi-locus index of association analysis was performed to assess the non-random association of all microsatellite loci in the dataset. The statistical significance of linkage disequilibria (LD) was tested using 10,000 Monte Carlo simulations. Significant multi-locus LD was observed in both symptomatic and asymptomatic malaria infections (*p* < 0.01) (Table 4). The LD ranged from 0.09 in symptomatic uncomplicated malaria to 0.12 in symptomatic complicated malaria infections.

**Table 4:** Linkage disequilibrium analysis for *P. falciparum* populations obtained in each infection category

Test factor	Asymptomatic	Symptomatic complicated	Symptomatic uncomplicated
V_D_	19.05	19.38	17.28
V_E_	4.95	4.46	4.74
I ^A^_S_	0.11	0.12	0.09
Var (V_D_)	0.25	0.27	0.18
P. value	< 0.01	< 0.01	< 0.01

The pairwise Wright’s fixation index (F_ST_) between the asymptomatic, symptomatic complicated and uncomplicated malaria infections revealed low genetic differentiation with F_ST_ values ranging between 0.0034 and 0.0105 (Table 5)

*V*_*D*_ variance, *V*_*E*_ expected variance if linkage equilibrium exists, *I*^*A*^_*S*_ standardized index of association

**Table 5:** Pairwise genetic differentiation (F_ST_) among malaria infection categories

	Asymptomatic	Symptomatic complicated	Symptomatic uncomplicated
Asymptomatic	0	0.0034	0.0059
Symptomatic complicated	0.0034	0	0.0034
Symptomatic uncomplicated	0.0059	0.0105	0

## Discussion

Genetically diverse and multiple *P. falciparum* malaria infections occur in both symptomatic and asymptomatic individuals within a population [42, 44]. The presence of diverse *P. falciparum* strains in asymptomatic individuals presents a major challenge to malaria elimination [46]. This is because asymptomatic infections often go undetected by conventional screening programs and remain untreated, persisting for over a year in an individual and thus serving as reservoirs for malaria parasites [47, 63]. Analyzing the genetic profiles of *P. falciparum* provides valuable information on malaria infection outcomes [64], helping to elucidate why some patients develop severe disease while others experience a milder form. It also offers essential parasite characteristics required for designing effective intervention strategies to control or prevent the disease [65].

Compared with other microsatellites, C2M34–313, Poly-α, and TA1 were found to be genetically more diverse, supporting the findings of a previous study by Ishengoma et al. [55] in Tanzania. This suggests that in malaria-endemic countries, C2M34–313, Poly-α, and TA1 may circulate at higher frequencies. The high polymorphism of these microsatellites reflects the high genetic diversity of *P. falciparum* parasites in the population [54, 55].

In this study, *P. falciparum* genetic diversity was high in both asymptomatic and symptomatic malaria-infected individuals, although it was slightly greater in asymptomatic individuals (He = 0.81 ± 0.15; Ne = 7.42 ± 5.15) compared to those with symptomatic uncomplicated malaria (He = 0.79 ± 0.17; Ne = 7.03 ± 4.28) and symptomatic complicated malaria (He = 0.80 ± 0.15; Ne = 6.07 ± 3.65). This finding suggests that asymptomatic malaria patients are likely to present a broader range of parasite genotypes than symptomatic malaria patients. Similar observations of high *P. falciparum* genetic diversity, characterized by high allele frequencies, have been reported in asymptomatic compared with symptomatic malaria individuals in Côte d'Ivoire [66]. Additionally, high *P. falciparum* genetic diversity, as indicated by a mean expected heterozygosity (He) of 0.81 (range: 0.57–0.95), has previously been noted among children with asymptomatic malaria infections in Kenya [43]. Other studies have indicated that symptomatic malaria infections can also harbor highly diverse *P. falciparum* infections [48, 67, 68]. Conversely, some studies have not reported a difference in *P. falciparum* genetic diversity between symptomatic and asymptomatic malaria infections [69, 70].

In symptomatic uncomplicated malaria infections, the high genetic diversity of *P. falciparum* may significantly affect disease severity and contribute to within-host competition among diverse strains [71]*.* These competitive interactions can lead to the selection of resistant genotypes [72]. The occurrence of high *P. falciparum* genetic diversity in asymptomatic infections may stem from two causes. First, in areas with moderate to high malaria transmission, the development of strain-specific immunity may favor asymptomatic carriers as reservoirs for parasite transmission, allowing the continuous introduction of new strains into the host population and thereby promoting genetic diversity and increased parasite fitness [3]. Second, asymptomatic infections often persist as low-density chronic infections, which can sustain diverse parasite strains within the host [73, 74].

The *P. falciparum* mean MOI did not significantly differ between asymptomatic and symptomatic infections, although it was slightly higher among asymptomatic individuals. This finding aligns with several previous studies reporting no significant differences in mean MOI between symptomatic and asymptomatic malaria individuals [45, 66] or between uncomplicated and complicated malaria cases [64]. Conversely, other studies have reported higher MOIs in asymptomatic malaria individuals [75]. For example, a recent study by Sarah-Matio, Elangwe et al*.* [42], conducted in a high malaria transmission area in Cameroon, revealed a greater MOI in asymptomatic individuals compared to symptomatic individuals (MOI = 5 in asymptomatic individuals versus median MOI = 2 in symptomatic individuals; *p* < 0.001). However, Simpson et al*.* [40] reported a contrasting result, with a higher MOI in symptomatic individuals than in asymptomatic malaria-infected individuals (2.24 versus 1.69; 95% CI 0.01–0.72; *p* = 0.046). The high mean MOI in symptomatic *P. falciparum* individuals may be partly due to the fact that symptomatic malaria infections often involve higher parasite densities [76], which can facilitate the presence of multiple strains. Infection with multiple strains in symptomatic individuals can lead to increased severity, complicating clinical management and treatment strategies [49]. Asymptomatic malaria cases often occur in areas with relatively high transmission intensities [77, 78], where multigenotype infections result from exposure to multiple parasite strains from different mosquito bites (superinfection) or from a single mosquito carrying multiple strains (co-transmission) [12, 13]. Additionally, asymptomatic malaria infections may be associated with partial immunity [63, 79], allowing a wider range of parasite strains to establish infection simultaneously. The presence of multiple parasite strains in asymptomatic malaria infections is thought to pose a risk for developing symptomatic malaria [75, 80], in addition to providing a reservoir of genetically diverse parasites [42, 70].

Our study revealed low linkage disequilibrium (LD) values (0.09 to 0.12), suggesting that alleles at different loci within the populations are largely independent. High malaria transmission areas favor high genetic recombination rates, which leads to low LD [81]. Additionally, the study observed similar allele frequencies in both asymptomatic and symptomatic malaria infections, as evidenced by low F_ST_ values ranging from 0.0034 to 0.0105. The low F_ST_ observed in our study is consistent with findings of low *P. falciparum* population genetic differentiation reported in other malaria-endemic countries [82, 83]. This indicates low genetic differentiation and, hence, relatively low overall genetic variation between the parasite populations. These results suggest relatively free gene flow across the study areas. In addition to reducing the occurrence of clinical disease, increased genetic diversity within a parasite population raises the risk of antimalarial drug resistance and could lower the efficacy of malaria vaccines [28, 84, 85].

Significant differences were observed in parasite density and hemoglobin (Hb) levels across individuals with asymptomatic and symptomatic malaria (*p* < 0.001). Symptomatic uncomplicated and complicated malaria patients had higher parasite densities and lower Hb levels compared to asymptomatic malaria patients. Increased *P. falciparum* parasite density has been reported in complicated malaria cases in previous studies [86, 87], whereas low parasitemia has been observed in asymptomatic individuals [88]. However, some studies have not reported significant differences in parasite density between asymptomatic and symptomatic malaria infections [89]. Asymptomatic individuals tend to have partial immunity through repeated exposure, allowing them to control parasite levels without experiencing symptoms [17]. The reduced parasite density among asymptomatic individuals enables them to maintain higher Hb levels compared to symptomatic malaria-infected individuals, where high parasite density leads to increased hemolysis [90]. High parasite density associated with inadequate dosing poses a risk for the development and emergence of antimalarial resistance [91]. Additionally, individuals with asymptomatic malaria often do not seek antimalarial treatment, allowing their infections to persist for extended periods and serve as reservoirs for malaria transmission [92–94]. Thus, malaria control programs should design strategies to eliminate asymptomatic malaria infections.

This current study provides new insights into the genetic diversity of *P. falciparum* among asymptomatic and symptomatic individuals, shedding light on aspects that had not been previously described. The findings highlight the complexity of malaria infections and their implications for disease management and control strategies, including the risk of antimalarial drug resistance. The high genetic diversity and MOI observed may challenge malaria vaccine development by potentially reducing vaccine efficacy, emphasizing the need for region-specific considerations.

However, there are some limitations to this study. Genotyping based on a limited set of microsatellite markers may have led to an overestimation of *P. falciparum* genetic diversity and MOI, as these markers may not fully represent the genome-wide genetic diversity. Employing a broader array of genetic markers or whole-genome sequencing could offer a more comprehensive evaluation of genetic diversity. Additionally, the study included relatively few complicated malaria cases, and the majority of participants were children (< 11 years), which may have influenced the accuracy of the assessments of genetic diversity and MOI in this group.

## Conclusion

This study found significant genetic diversity among *P. falciparum* parasites associated with different infection categories with asymptomatic individuals exhibiting greater genetic diversity. The high genetic diversity and evolving parasite populations highlight the need for strategies to maximize the effectiveness of malaria vaccines. Future studies should employ a longitudinal study to assess temporal and seasonality changes in *P. falciparum* genetic diversity and MOI.

## Supplementary Information

Additional file 1.

Additional file 2.

Additional file 3.

## Data Availability

The detailed datasets used during the current study are available from the corresponding author upon request. Genotyping raw data are provided in Additional File 3 (Table S3).

## Abbreviations

DBS: Dried blood spot
Hb: Hemoglobin
He: Expected heterozygosity
LD: Linkage disequilibrium
MOI: Multiplicity of infection
MSP: Merozoite surface protein
PBS: Phosphate-buffered saline
PCR: Polymerase chain reaction
*PfEMP1*: *P. falciparum* Erythrocyte membrane protein 1
PRISM: Program for Resistance, Immunology and Modeling of Malaria
RFU: Relative fluorescence units
