# Supplementary material for: Plasmodium falciparum genetic diversity and multiplicity of infection among asymptomatic and symptomatic malaria-infected individuals in Uganda
Source: Trop Med Health. 2024 Nov 14;52:86. doi: 10.1186/s41182-024-00656-7 (PMC11562702; doi:10.1186/s41182-024-00656-7)
Supplement: Supplementary file 1 — Additional file 1. [file 41182_2024_656_MOESM1_ESM.doc]

*Plasmodium falciparum* genetic diversity and multiplicity of infection among asymptomatic and symptomatic malaria-infected individuals in Uganda

**
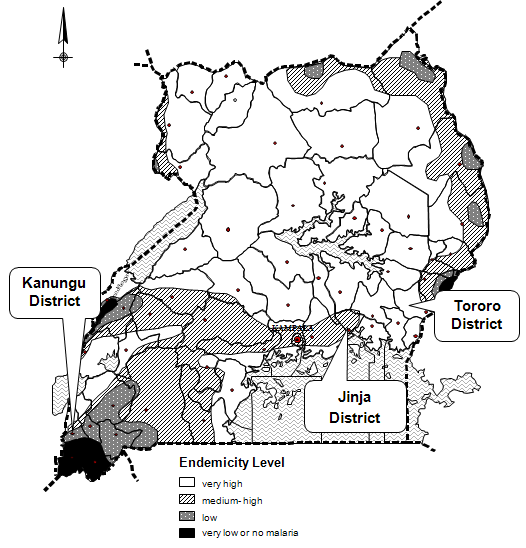
**

**Fig.1** Map of Uganda showing malaria endemicity of the study sites at the time of sample collection
